# Psycho-Oncology: A Bibliometric Review of the 100 Most-Cited Articles

**DOI:** 10.3390/healthcare9081008

**Published:** 2021-08-06

**Authors:** Susan Fox, Julie Lynch, Paul D’Alton, Alan Carr

**Affiliations:** 1 School of Psychology, University College Dublin, D04 V1W8 Dublin, Ireland; julie.lynch@ucdconnect.ie (J.L.); paul.dalton@ucd.ie (P.D.); alan.carr@ucd.ie (A.C.); 2 St Vincent’s University Hospital, D04 T6F4 Dublin, Ireland

**Keywords:** cancer, bibliometrics, psycho-oncology, multidisciplinary, oncology, review

## Abstract

(1) Background: A bibliometric review of psycho-oncology research is overdue. (2) Methods: The 100 most-cited journal articles were compiled and ranked according to Scopus. (3) Results: The total citation count for the results ranged from 488–8509 (Mean = 940.27; *SD* = 1015.69). A significant correlation was found between years since publication and number of citations (*p* = 0.039). The majority of research originated from the United States (66%). The vast majority of research publications were original articles (80%). Observational research study designs represented the majority of studies (37%). Mixed cancer population research studies represented the largest cancer research population (36%). Positive psychology topics represented the most prolific proportion of studies (30%). Findings were reported in line with PRISMA-ScR guidelines. (4) Conclusions: This analysis offers a comprehensive account of seminal journal articles in psycho-oncology, identifying landmark contributions and areas for future research developments within the field, namely highlighting a need for more RCT studies. This analysis serves as an educational tool for interdisciplinary researchers and clinicians to support compassionate cancer care.

## 1. Introduction

Psycho-oncology is a collaborative, cross-disciplinary subspecialty of oncology with domains in the psychological, social, behavioural, and ethical aspects of cancer in clinical care [1,2]. The discipline provides clinical and research material about issues clinically relevant to health professionals who provide psychosocial services to cancer patients, their families, and their caregivers [3]. The foundations of the field first came into existence in the 1970s [1], the evolution of which has previously been detailed by the founder of the field, Dr Jimmie Holland [1,3].

Overtime a large body of literature has been published comprising a wide range of relevant research and clinical themes. A previous review of this wealth of literature by Greer outlines the important need to “close the yawning gap between current knowledge and therapeutic skills on the one hand and actual clinical care of cancer patients on the other” [4]. As the discipline approaches fifty years since formal foundation, a bibliometric review of the literature is warranted to aid the synthesis and implementation of the evidence base.

Citation count is an important metric in understanding the significance of a research contribution to a research field [5,6,7]. Situational analyses which identify research that has exerted significant citation influence offers researchers and clinicians an introduction to seminal research publications. It can be argued that the most-cited publications of a research field theoretically contribute the most to the respective field [8,9,10,11]. Notably, the approach has proved useful in practice-driven research funding decision-making by offering objective and reliable bibliometric quantitative analysis of grant productivity [8,12]. Bibliometric analyses with the aid of bibliographic electronic databases offer a systematic overview of peer-reviewed research in a range of disciplines and research fields [13,14]. Neurosciences have widely adopted the methodology to identify seminal research and contributors [15,16,17,18,19,20,21,22]. The use of bibliometric methodologies is emerging in cancer care [12,23,24,25,26,27,28,29]. However, to date, no known research has identified the highest-cited articles in psycho-oncology. Therefore, the aim of this study was to identify and describe the characteristics of seminal journal articles that have contributed to the development of the field of psycho-oncology. Given the extensive remit of the multidisciplinary field of psycho-oncology, a bibliometric review of the literature may prove a helpful introduction for researchers and clinicians working in cancer care.

## 2. Materials and Methods

### 2.1. Study Design

This article describes a citation analysis of journal articles in the field of psycho-oncology pertaining to the guidance of clinical practice and research. A review of the 100 most-cited papers is consistent with the methodological approach to bibliometric reviews in health research [13,14,16,17,30,31,32,33]. A review protocol was developed to support the study’s objectives, search strategy, inclusion/exclusion criteria and risk of bias assessment. No deviations from the protocol were made.

### 2.2. Search Strategy and Study Selection

Publications pertaining to “psychosocial oncology” or “psycho-oncology” and psychological processes relating to cancer were identified. The search strategy was informed by the keywords and terms constructed by key journals in the field [2].

A time limit of publications from 1970 to 2020 was imposed on the searches, as the field of psycho-oncology was formally founded in the mid-1970s [1]. Searches were limited to the English language due to resource limitations (see Supplementary Material for detailed search strategy). The list of the top 100 cited articles was compiled and ranked according to the outputs from the Scopus database search in March 2020. Scopus was selected as the primary database because it provides access to more journals (approximately 34,346 peer-reviewed journals) than other widely used electronic databases, such as Web of Science (approximately 24,748 peer-reviewed journals) [34]. Key to database selection, Scopus provides tools for citation overview, allowing for bibliometric ranking of credited citations. Furthermore, several key psychosocial-oncology journals are indexed within Scopus. Inclusion of one electronic database is standard practice in bibliometric analyses [14,16,17,30,31,33]. Results retrieved from Scopus were sorted using the sorting option “times cited—highest to lowest.” Scopus outputs were then exported to Covidence, an electronic primary screening and data extraction tool, which has been recommended as best practice in rigorous review methodology data charting [35,36,37]. Duplicates were removed. Two reviewers (S.F. and J.L.) independently applied the inclusion and exclusion criteria to screen each title and abstract using the Covidence platform. Disagreements between the two reviewers were resolved through a further detailed review of the article(s) in question, and discussion until consensus was reached. An equivalent process of review was conducted for the full-text screening phase. Cohen’s κ indicated almost perfect interrater reliability (κ = 0.97, 98.87% of agreement).

### 2.3. Eligibility Criteria

Eligibility criteria were bound to the remit outlined in key definitions of psycho-oncology [1,2,38]. Journal articles were eligible for inclusion if their major focus addressed the psychological, social, behavioural, ethical, and systemic dimensions of cancer (including stable and modifiable confounding and interacting factors); specifically, the psychological responses of patients to cancer at all stages of the disease, and that of their families and caregivers including their health professionals; and the factors that may influence the disease process [1]. Given the intrinsic multidisciplinary nature of psycho-oncology, journal articles from a range of disciplines were eligible for inclusion, where the primary focus explored subjects within the defined remit of psycho-oncology. Eligibility criteria were extended to counselling, education, epidemiology, health advocacy, neurology, nursing, nutrition, palliative care, physical therapy, psychiatry, psychology, public health, social work, sociology, and oncology specialities [2]. Journal articles published in the English language were eligible for inclusion. No restrictions were placed on the type of research model, article type (e.g., research article, review, conference proceedings, editorial, letter, etc.).

Studies were ineligible for inclusion if they were the following:

1. Journal articles with primarily medical foci despite the inclusion of brief quality of life measures;
2. Journal articles which described mixed patient populations beyond oncology; or
3. Did not have psycho-oncology research or practice as key foci.

### 2.4. Data Extraction

Data were extracted independently by the two reviewers. Information was extracted on the following variables: (1) title; (2) authorship and publication year; (3) country of publication and first author’s affiliation at the time of publication; (4) journal; (5) article type (e.g., intervention, systematic review); (6) article global subject (e.g., cancer prevention, psychoneuroimmunology or post-traumatic growth); (7) number of citations; (8) and citation rank. High percentage agreement between raters was found (percentage agreement = 94%).

### 2.5. Self-Citations

Using the “exclude self-citations” tool in Scopus, the percentage of self-citations within the list of 100 most highly cited articles derived from Scopus was calculated.

### 2.6. Statistical Analysis

The Pearson correlation coefficient (*r*) was calculated to determine whether the number of years since publication was correlated with total number of citations among the included articles.

### 2.7. Publication Trends

Additional searches using the terms “psycho-oncology” and “psychosocial oncology” were conducted within Scopus. These searches and the resulting data provide a broad overview of the publication trends of articles using these terms.

## 3. Results

### 3.1. Study Selection

**Figure 1:**
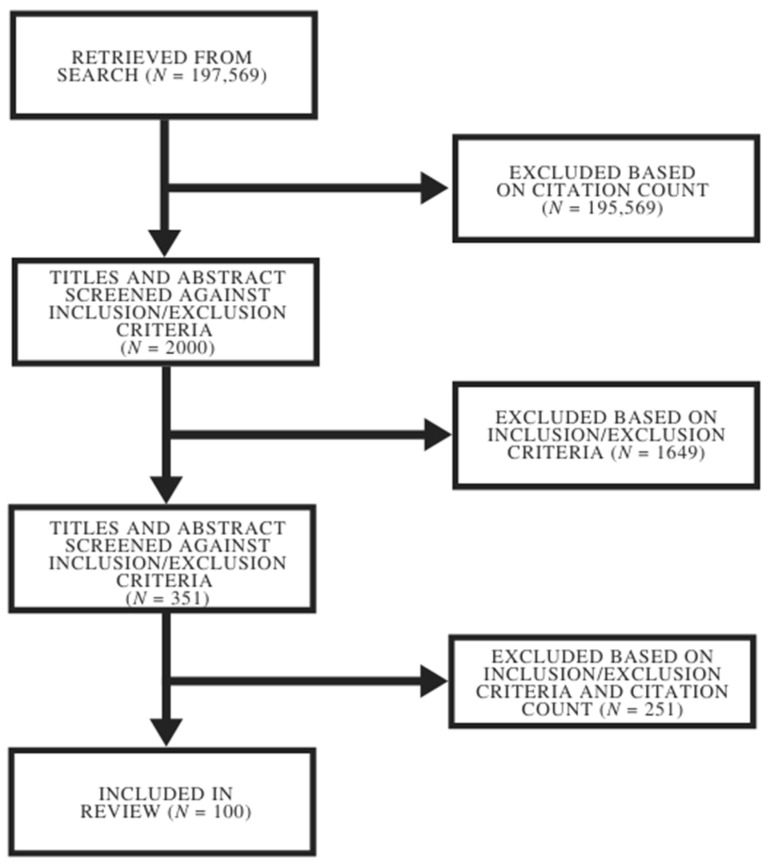
The PRISMA flowchart of study selection.

A PRISMA flow diagram for the Scopus results is provided in Figure 1. The initial search returned 197,569 results, of which the titles and abstracts of the 2000 highest-cited articles were screened using the eligibility criteria. Full-text screening was completed for 351 articles. Results were ranked according to citation counts to represent the 100 most-cited articles. A table of the included 100 publications and a citation details are presented in the Appendix A.

### 3.2. Self-Citations

Self-citations were found to represent 4.4 per cent of total citations retrieved from Scopus.

### 3.3. Study Characteristics

**Figure 2:**
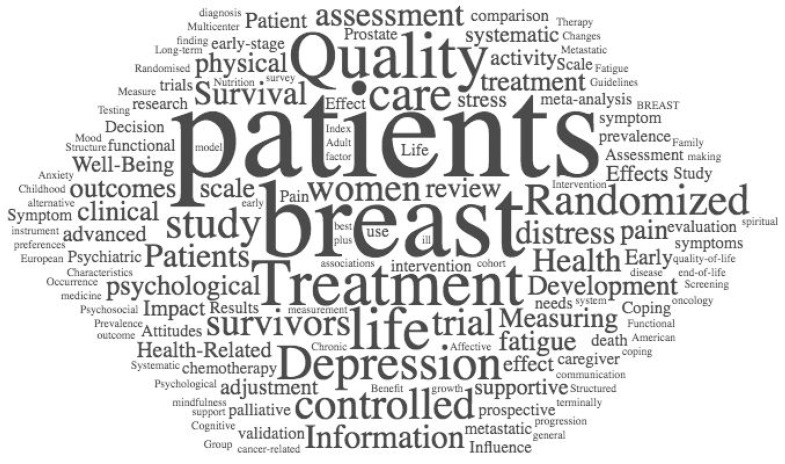
Word cloud of the words used in the titles of the 100 included studies.

The characteristics of the articles retrieved are provided in Table 1. Of the 100 included papers, the highest-cited articles were published between 1992 and 2005. A significant correlation was found between years since publication and the number of citations (*p* = 0.039). The citation range was 488–8509 (mean = 940.27, *SD* = 1015.69). Similarly to recent bibliometric reviews [22,39] a word cloud of the words contained in the titles of the 100 included studies was generated using wordle.net in order to depict influential prevailing words and themes within the field of psycho-oncology. Popular words and phrases are highlighted based on frequency and relevance to the titles of the 100 included papers (see Figure 2).

### 3.4. The 100 Most-Cited Articles

The distribution of results for the 100 most-cited articles is presented in Table 1. A comprehensive list of results is presented in the Appendix A.

### 3.5. Country of Publication

The country of origin of the first author for each article represented study origin data. Overall, 10 nations contributed to included study origin. The United States of America represented the largest contribution of studies (66%), followed respectively by the United Kingdom (12%) and Canada (10%). See all contributory countries in Table 1, panel 1.

### 3.6. Publication Type

The distribution of document type is presented in panel 2. Original articles represented 80% of the studies. Review papers and conference papers represented 18% and 2% of studies respectively.

### 3.7. Type of Study

Observational research study designs represented the majority of studies (37%). Cross-sectional observational designs represented the largest cohort of studies (20%) followed closely by prospective designs (17%). Tool development/evaluation, intervention and review studies each represented 21% of studies. Comprehensive distribution of study methodology is presented in panel 3.

### 3.8. Global Subject Topic

Positive psychology represented the largest proportion of included studies (30%), where the overarching global subject topic of studies examined psychological well-being and post-traumatic growth (14%), quality of life (14%), and mindfulness (2%). Clinical psychology global topics represented the second-largest cohort of studies (12%), where topics included psychological distress and mental health outcome including depression and suicidality were explored. Parallel psychological and physical health outcomes were the global subject topic for 2% of studies. Symptom prevalence represented 15% of study global subject topics, where 5% of studies examined pain prevalence and 10% explored additional cancer-related sequelae including the prevalence of cancer-related fatigue. Health promotion studies represented 6% of studies. These studies explored cancer prevention including self-monitoring behaviour, genomic testing, and survivorship intervention studies. Patient–physician communication and patient communication needs represented 10% of studies. Patient treatment choices including complementary and alternative medicine (CAM) represented 4% of studies. Palliative or supportive care studies represented 6%. Psychoneuroimmunology research represented 6% of the studies. Survivorship analyses represented 6% of the studies. Family system outcomes represented 1% of studies. See panel 4 for comprehensive results.

### 3.9. Cancer Population

The largest proportion of studies explored mixed cancer populations (36%) followed by breast cancer populations (25%), and advanced/terminal cancer populations (15%). A significant proportion of studies did not define the cancer population (13%). A further 5% of studies included prostate cancer patients. Lung and malignant melanoma patients each represented 2% of studies. Brain, cervical, gastric, laryngeal, and colorectal cancer populations each represented 1% of studies. See panel 5 for comprehensive results. The vast majority of the included studies examined adult populations (97%). The remaining 3% of the studies examined child populations.

### 3.10. Major Contributing Journals and Periods

The 100 most-cited articles were published in 46 journals; 17 journals represented more than one study. The major contributing journals are presented in Table 2. The journals that contributed six or more of the 100 most-cited studies included the *Journal of the American Medical Association,* *The New England Journal of Medicine, The Lancet, The British Medical Journal, Health Psychology* and the *Journal of Clinical Oncology*. The journal that published the 100 most-cited psycho-oncology studies with the highest citation count was *The Lancet*.

**Figure 3:**
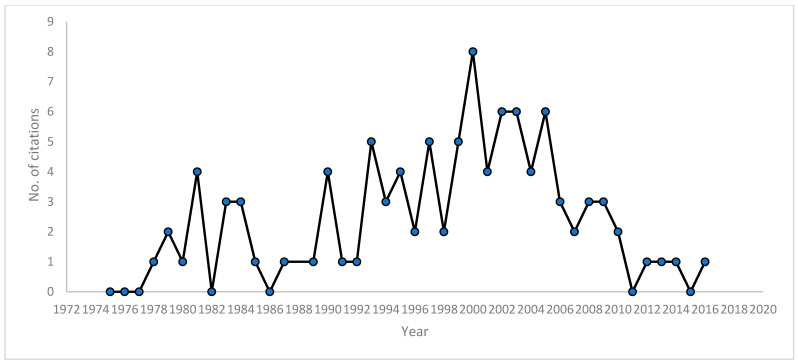
Publication trends for the 100 included publications.

The 100 most-cited studies were published from 1975 to 2016. Figure 3 presents the publication trends for the 100 included publications. A period of 24 years represented 79% of studies, where the majority of studies were published between 1981–2005. A peak in publications was observed in the year 2000.

**Figure 4:**
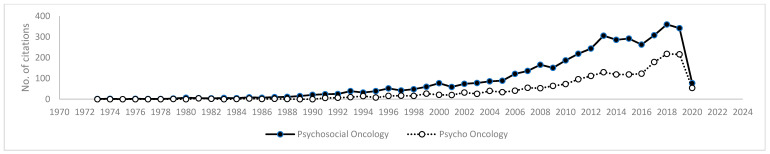
Publications by year for the terms “psychosocial oncology” and “psycho-oncology” in Scopus.

Figure 4 provides an overview of publication trends within Scopus under the key terms “psycho oncology” and “psychosocial oncology”. Publications under the term “psychosocial oncology” precede “psycho oncology” publications commencing in 1973. The term “psycho oncology” presents initially in 1979, demonstrating the evolution of the discipline. A peak in publications was observed in 2018 for both search terms.

### 3.11. The 10 Most-Cited Articles

The ten most-cited studies are presented in Table 3. The articles included the following: three studies detailing the development and assessment of psychometric measures, two quality of life measures [40,41] and one pain measure [42]; two reviews, the first explores psychological adjustment to breast cancer diagnosis [43], and the latter explores the role of mindfulness in psychological well-being and includes a prospective mindfulness-based intervention for early-stage cancer patients [44]; one cross-sectional observational study which compares psychological distress prevalence by cancer site [45] two controlled trial studies, one randomised controlled trial exploring an early palliative care intervention for metastatic lung cancer patients [46]; and one prospective controlled trial exploring a psychosocial group-based intervention for metastatic breast cancer patients [47]; and finally two prospective cohort studies, one identifying the determinants of quality of life and satisfaction among prostate cancer survivors [48]; and one determining the impact of end-of-life patient–physician communication on patient mental health, medical care near death, and caregiver bereavement adjustment in advanced cancer patients and their family systems [49].

### 3.12. Major Contributing Authors

Overall, a total of 158 authors contributed to the results. There was wide, disparate authorship for first authors where 91 first authors represented the 100 included studies. Of these included studies, only one first author had published three studies as first author [47,50,51]. Three other first authors each published two studies as first author [40,52,53,54,55,56]. Each of these authors contributed as co-authors to other studies indicating a psycho-oncological focus in their published work. Cella, D. contributed the largest number of studies to the research (*n* = 7) [40,52,57,58,59,60,61]. Table 4 presents results for authors who contributed three or more of the 100 most-cited psycho-oncology articles.

## 4. Discussion

The aim of this review was to perform a bibliometric analysis of the 100 most-cited journal articles in psycho-oncology. It is, to the best of our knowledge, the first study to identify and describe the characteristics of highly cited journal articles and publication trends that have contributed to the development of the field.

The results of the bibliometric review provide a systematic overview of seminal research in psycho-oncology overtime. Our review presents a body of evidence which may have multiple applications for researchers and clinicians alike working in the field of psycho-oncology, including potential for the development of educational materials, journal editorial strategy, and future research.

In accordance with Scopus, our analysis revealed that the 100 most-cited articles were published between 1975 and 2016. This finding is in keeping with the timeline of previous reviews which describe the evolution of the discipline, from a time when a diagnosis of cancer was stigmatised and not openly disclosed to patients, and towards a time of more trauma-informed cancer care [1,2,38]. The 100 most-cited journal articles were all published by 2016 and the ten most-cited articles averaged 21.8 years since publication, indicating that the research exists along a developmental trajectory whereby time impacts on citation count and subsequent influence. The majority of research originated from the United States (66%). The vast majority of research publications were original articles (80%). Observational research study designs represented the majority of studies (37%). Mixed cancer population research studies represented the largest cancer research population (36%).

Our analysis revealed that positive psychology topics and clinical psychosocial-oncology topics represented the most prolific proportion of included studies. This finding reflects one of the most fundamental questions that psycho-oncology seeks to understand—how do people with cancer feel? The global subject topics included in our analysis reflect the targets of previous narrative reviews of psycho-oncology [38]. Other subject topics included in our review explored parallel psychological and physical health outcomes, symptom prevalence including pain and cancer-related fatigue, health promotion and cancer prevention research including self-monitoring behaviour, genomic testing and survivorship intervention studies, patient–physician communication and patient communication needs, patient treatment choices including complementary and alternative medicine, palliative care research, psychoneuroimmunology, survivorship, and family system outcomes. Our analysis highlights the psychosocial transitory nature of cancer, which presents the potential for both positive and negative outcomes [62]. Findings reflect increased recognition for the “people part” of cancer care and the sixth vital sign in medicine—distress [63]. Enhanced patient participation and increased patient–physician communication in treatment decisions have been described in recent reviews of the field [3]. Beyond this, the analysis emphasises the impact of psychosocial factors in physical health and the growing attention that psychoneuroimmunology research has gained [64]. A paucity of highly cited research on adherance to cancer treatment was identified. Given the value of research on this subject topic for MDTs, this factor represents a deficit among included study topics.

Journal and author contributions were widely heterogeneous in nature. Our analyses revealed 91 first authors contributors across the 100 included studies. Notably, self-citations represented a very small percentage of citations (4.4%). A previous review of self-citations in research indicated that self-citations typically account for an average 10–20% of citation counts [65]. The 100 included articles were published in 46 journals, where 17 journals represented more than one study. Included studies were published in high-impact factor journals. Our analysis of highly cited journal articles reflects the interdisciplinary nature of psychosocial-oncology, which demonstrated the interfacing and overlapping boundaries with general medicine, oncology, psychiatry, pain medicine, health, and social psychology [2]. In keeping with this finding, interdisciplinary researcher and Chair of the Interdisciplinary Department of Medical Social Sciences at Northwestern University, Prof. David Cella, was the most prolific author) [40,52,57,58,59,60,61]. Additional analysis of global publication trends within Scopus indicated that the term “psychosocial oncology” precedes “psycho-oncology”. Although the percentage of publication increased over time, a noticeable peak in publications was observed in 2018 for both search terms, clearly demonstrating the dynamic evolution of the discipline. In addition to time, other secular trends such as increased capacity of search engines and access to research articles online positively impact citation count.

### 4.1. Strengths and Limitations

Beyond its novel contribution, this bibliometric analysis was strengthened by the use of two search methods. The keyword search enabled the identification of publication trends for psychosocial oncology in addition to psycho-oncology. This methodological consideration enriches the tapestry of the findings as psychosocial oncology terminology precedes psycho-oncology in the evolution of the field [1]. Additionally, the review was strengthened by its adherence to bibliometric technical methods [66,67]. A further strength of this study is the assessment of the prevalence of self-citations. Inclusion of this analyses explores academic biases which can artificially inflate citation impact rate by objectively assessing the impact of ‘other-driven’ citations [5,68]. Inversely, this bibliometric review is not without its own limitations. Specifically, a publication bias may have been induced by the methodological limitation to only include English language publications. This limitation may explain why the study origins of the leading contributing counties were Anglophonic countries, namely the USA, UK, and Canada, because seminal articles in other languages were not included.

Though comprehensive, our analysis was limited in that research influence was operationalised using a citation-driven approach. Indeed each metric has its own limitations that need to be considered when selecting an appropriate metric for evaluation. Given the advantages and disadvantages of citation counts, our analysis should be interpreted with caution [5,68]. In academia, it is a common misconception that citation counts provide a benchmark for the impact of research. It should be noted that citation-driven bibliometric analyses neglect to consider the influence of landmark conceptual research journal articles. Further, our analysis does not assess the quality of the research presented. Quality appraisal of the findings was not possible, given the heterogeneity of the resulting output. It is important to consider that citation count fails to represent the quality of the research. Our analysis cannot identify with any authority the key conceptual journal articles that have shaped the trajectory and development of the field. This shortcoming serves as a rationale to support the investigation of conceptually-driven influential psychosocial-oncology research in future. However, it can be noted that previous review articles have reflected on key conceptual developments [1,3]. Finally, the search was limited to the Scopus electronic database. While the inclusion of one electronic database is standard practice in bibliometric analyses [14,16,17,30,31,33], it is important to critique any outcome metric provider. Key to database selection, Scopus provides tools for citation overview including self-citation analysis. However, highly cited articles in journals not indexed in Scopus may not have been captured in the findings. Further, citation count varies between databases [5,68]. For this reason, the ranking of included articles should be interpreted with caution.

### 4.2. Implications for Psycho-Oncology Practice and Research

Given the extensive remit of the multidisciplinary field of oncology, a bibliometric review of the psycho-oncology literature may prove a helpful introduction for multidisciplinary teams working in cancer care. This review offers a broad overview of seminal research in the field. It also honours the key contributors to the field by identifying work that has been frequently cited by other researchers. Clinicians new to the field may perceive psycho-oncology to solely encompass the psychological health of oncology patients. It is important to educate new clinicians to routinely and sensitively consider the individual and systemic level psychological, social, behavioural, and ethical aspects of cancer, since they can substantially influence the outcome of treatment. This review provides health professions with an educational resource that compounds our understanding of the mind–body interaction that continues to challenge a mechanical model of cancer.

This study generates knowledge regarding the intricacies of psycho-oncology clinical practice and research work and emphasises the need for compassionate collaborative, cross-disciplinary cancer care. It is important to acknowledge the need for translation beyond citation into interdisciplinary practice.

### 4.3. Future Directions

This bibliometric review provides a situational analysis of the field of psycho-oncology in the present, as opposed to a view of the future of the field. As discussed previously, it is important to note that our analyses offers a snapshot of highly cited seminal research in psycho-oncology at one point in time. Our analysis is best viewed as a live document responding to the evolving priorities of the field. We recommend replication studies at regular intervals to update the findings in order to maximise educational value. The nature of our review offers a broad scope of the field; future research could consider a more introspective bibliometric analysis of *Psycho-Oncology* exclusively [69]. Bibliometric reviews of single journals help chart the developmental growth and trajectory of a journal by identifying research trends, areas of research neglect, and disparities in academic publishing. Findings may offer editorial boards insight to help close gaps in research and help support funded external research grant calls [69].

Such an approach would offer increased insight and further support the maturity of the discipline, educational materials, and journal editorial strategy.

Relatively few high-quality RCT studies were included. This deficit identified in our analysis generates greater understanding of one of the pervading gaps in the research field. Our analysis underscores the critical need to enhance the science of psycho-oncology. Greater emphasis on high-quality methodological research is needed. This finding serves as a specific area of research opportunity to greater align future research to the needs of the field.

## 5. Conclusions

Psycho-oncology is a vast subspecialty of oncology encompassing diverse areas of clinical practice and research, focusing on the humanism in cancer-prevention, treatment, and aftercare. Given the evolution of the field from a place where the word ‘cancer’ was stigmatised and the feelings of cancer patients were not acknowledged, a bibliometric review which reflects on almost fifty years since the formal foundation of the field is overdue. This bibliometric review identifies the most frequently cited psycho-oncology journal articles published across all journals listed in Scopus. The results identified in this study are landmark papers that have contributed greatly to the field. This review denotes the growing nature of the discipline, which continues to advance. As the discipline has become increasingly established, there has been a simultaneous increase in research publications. While this study is not without its limitations, it is hoped that identification of seminal research publications will help inform future research contributions. This analysis should serve to support the routine consideration of the psychosocial aspects of cancer care. It may provide a useful educational tool for interdisciplinary clinicians. It is hoped that it will encourage considered compassionate care for cancer patients.

## Figures and Tables

**Table 1:** Study characteristics of the top 100 published articles.

Study Characteristics	Frequency	Citations	
	(%)	Mean ± SD	Range
1. Country of Origin			
United States	66	966.83 ± 806.81	491–4667
United Kingdom	12	635 ± 87.44	518–801
Canada	10	667 ± 132.54	512–909
The Netherlands	4	2615.75 ± 3954.77	541–8547
Germany	2	768.5 ± 102.53	696–841
Sweden	2	627.5 ± 75.66	574–681
Australia	1	-	1159
Austria	1	-	605
Brazil	1	-	723
Denmark	1	-	532
2. Publication Type			
Article	80	925.23 ± 992.84	486–8451
Review paper	18	1044.5 ± 1184.88	500–4565
Conference paper	2	600 ± 97.58	531–669
3. Study Type			
Tool development/evaluation	21	1347.10 ± 1811.02	505–8451
Observational (cross-sectional)	20	782.05 ± 287.63	500–1369
Observational (prospective cohort)	17	716.35 ± 303.22	488–1549
Review (non-systematic)	12	1045.75 ± 1168.71	486–4565
Intervention (RCT)	11	906 ± 970.03	486–3824
Intervention (non-RCT)	10	903.5 ± 407.36	511–1782
Review (systematic/meta-analysis)	9	619.89 ± 96.27	507–767
4. Global Subject Topic			
Psychological well-being	14	758.93 ± 371.98	488–1862
Quality of life	14	1556 ± 2142.43	555–8451
Psychological distress/Mental health	12	892.33 ± 446.91	517–1782
Patient–physician communication	10	764.70 ± 287.31	507–1480
Symptom prevalence	10	672.80 ± 181.95	505–1079
Health promotion/Cancer prevention	6	754.83 ± 218.82	486–1153
Palliative/Supportive care	6	1193.50 ± 1295.41	511–3824
Psychoneuroimmunology	6	570.83 ± 79.56	504–715
Pain	5	1220.40 ± 944.66	583–2885
Patient treatment choices	4	573.75 ± 73.89	500–656
Mindfulness	2	2615 ± 2757.72	665–4565
Psychological and physical health outcomes	2	627.50 ± 77.01	573–682
Survivorship	2	696.50 ± 221.32	540–853
Family/system outcomes	1	-	566
5. Cancer Type			
Mixed cancer population	36	907.33 ± 832.36	486–4565
Breast	25	757.08 ± 369.68	488–1862
Advanced/terminal	15	768.92 ± 284.83	511–1480
Undefined	13	890.15 ± 621.50	507–2885
Prostate	5	916.2 ± 385.48	571–1549
Lung	2	6137.5 ± 3271.78	3824–8451
Malignant melanoma	2	682 ± 241.83	511–853
Brain	1	-	569
Cervical	1	-	580
Gastric	1	-	680
Laryngeal	1	-	500
Colorectal	1		603
6. Population			
Adult	97	944.14 ± 1021.61	486–8451
Child	3	653.33 ± 133.13	573–807

**Table 2:** Journals that have published the highest-cited articles as listed in Scopus.

Journal	Frequency (%)	Sum (No. Citations)	Mean ± SD (No. Citations)	Range (No. Citations)
Journal of the American Medical Association	15	12,823	854.87 ± 303.16	507–1480
New England Journal of Medicine	8	9213	1151.63 ± 1130.83	500–3824
Lancet	7	13,158	1879.71 ± 2932.52	511–8451
British Medical Journal	6	3773	628.83 ± 54.09	597–738
Health Psychology	6	3591	598.5 ± 85.11	500–695
Journal of Clinical Oncology	6	7056	1176 ± 1219.80	540–3655
Journal of Personality and Social Psychology	4	7038	1759.5 ± 1891.73	605–4565
Archives of General Psychiatry	3	2062	687.33 ± 171.25	511–853
Pain	3	2458	819.33 ± 227.14	583–1036
Annals of Oncology	2	1336	668 ± 16.97	656–680
Archives of Internal Medicine	2	1088	544 ± 26.87	525–563
Canadian Medical Association Journal	2	1247	623.5 ± 81.32	566–681
Cancer	2	1055	527.5 ± 28.99	507–548
Journal of Pain and Symptom Management	2	1616	808 ± 383.25	537–1079
Lancet Oncology	2	1284	642 ± 103.24	569–715
Psycho-Oncology	2	2166	1083 ± 746.70	555–1611
Seminars in Haematology	2	1200	600 ± 97.58	531–669
CA: Cancer Journal for Clinicians	2	1188	594 ± 152.74	486–702

**Table 3:** The 10 highest-cited publications in psycho-oncology.

Rank	Author and Year	Citations	Description
1	Aaronson et al. 1993	8451	An assessment of the EORTC QLQ-C30 quality of life psychometric tool.
2	Brown and Ryan 2003	4565	An overview of the role of mindfulness in psychological well-being and a prospective mindfulness-based intervention for early-stage cancer patients.
3	Temel et al. 2010	3824	An RCT where newly diagnosed patients with metastatic lung cancer were randomised to receive either early palliative care integrated with standard oncologic care/standard oncologic care.
4	Cella et al. 1993	3655	The development and assessment of the FACT quality of life psychometric tool.
5	Cleeland and Ryan 1994	2885	The development of the BPI pain psychometric tool.
6	Taylor 1983	1862	A review of psychological adjustment to breast cancer diagnosis.
7	Spiegel et al. 1989	1782	A prospective controlled trial where patients with metastatic breast cancer were randomised to psychosocial group-based intervention and standard oncologic care/standard oncologic care.
8	Zabora et al. 2001	1611	A cross-sectional observational study of psychological distress prevalence and comparison by cancer site.
9	Sanda et al. 2008	1549	A prospective cohort study identifying determinants of quality of life and satisfaction among prostate cancer survivors.
10	Wright et al. 2008	1480	A longitudinal prospective cohort study of patients with advanced cancer and families to determine the impact of end-of-life patient–physician communication on patient/family outcomes.

**Table 4:** Authors who contributed three or more of the 100 most-cited psycho-oncology articles.

Author	Total Articles (*n*)	Role of Author in Total Articles		Citation Count ± SD
		First and Corresponding Author	Co-Author	
Cella, D.	7	2	5	1162.29 ± 1115.27
Courneya, K.S.	5	0	5	635.2 ± 117.53
Breitbart, W.	4	1	3	664.5 ± 115.30
Litwin, M.S.	4	2	2	1002.5 ± 385.32
Portenoy, R.K.	4	2	2	761.75 ± 183.05
Demark-Wahnefried, W.	3	1	2	642.67 ± 137.00
Ganz, P.A.	3	0	3	740.67 ± 102.26
Greer, S.	3	1	2	534 ± 20.66
Sloan, J.A.	3	0	3	747.67 ± 173.62
Spiegel, D.	3	3	0	994.67 ± 688.72

## Data Availability

Data sharing is not applicable to this article as no new data were created or analysed in this study.

## Supplementary Materials

The following are available online at https://www.mdpi.com/article/10.3390/healthcare9081008/s1: Supplementary Material 1. Scopus String Search, Supplementary Material 2. Preferred Reporting Items for Systematic reviews and Meta-Analyses extension for Scoping Reviews (PRISMA-ScR) Checklist.

## Appendix A

**Table A1:** Comprehensive results from Scopus for the 100 most cited journal articles.

Rank	Authors	Title	Year	Journal
1	Aaronson N.K., et al. [41]	The European Organization For Research And Treatment Of Cancer QLQ-C30: A Quality-Of-Life Instrument For Use In International Clinical Trials In Oncology	1993	Journal of the National Cancer Institute
2	Brown K.W., & Ryan R.M. [44]	The Benefits Of Being Present: Mindfulness And Its Role In Psychological Well-Being	2003	Journal of Personality and Social Psychology
3	Temel J.S., et al. [46]	Early Palliative Care For Patients With Metastatic Non-Small-Cell Lung Cancer	2010	New England Journal of Medicine
4	Cella D.F., et al. [40]	The Functional Assessment Of Cancer Therapy Scale: Development And Validation Of The General Measure	1993	Journal of Clinical Oncology
5	Cleeland C.S., et al. [42]	Pain Assessment: Global Use Of The Brief Pain Inventory.	1994	Annals of the Academy of Medicine, Singapore
6	Taylor S.E. [43]	Adjustment To Threatening Events: A Theory Of Cognitive Adaptation	1983	American Psychologist
7	Spiegel D., et al. [47]	Effect Of Psychosocial Treatment On Survival Of Patients With Metastatic Breast Cancer	1989	The Lancet
8	Zabora J., et al. [45]	The Prevalence Of Psychological Distress By Cancer Site	2001	Psycho-Oncology
9	Sanda M.G., et al. [48]	Quality Of Life And Satisfaction With Outcome Among Prostate-Cancer Survivors	2008	New England Journal of Medicine
10	Wright A.A., et al. [49]	Associations Between End-Of-Life Discussions, Patient Mental Health, Medical Care Near Death, And Caregiver Bereavement Adjustment	2008	Journal of the American Medical Association
11	Derogatis L.R., et al. [70]	The Prevalence Of Psychiatric Disorders Among Cancer Patients	1983	Journal of the American Medical Association
12	Carver C.S., et al. [71]	How Coping Mediates The Effect Of Optimism On Distress: A Study Of Women With Early Stage Breast Cancer	1993	Journal of Personality and Social Psychology
13	Spitzer W.O., et al. [72]	Measuring The Quality Of Life Of Cancer Patients. A Concise QL-Index For Use By Physicians	1981	Journal of Chronic Diseases
14	Holmes M.D., et al. [73]	Physical Activity And Survival After Breast Cancer Diagnosis	2005	Journal of the American Medical Association
15	Murthy V.H., et al. [74]	Participation In Cancer Clinical Trials: Race-, Sex-, And Age-Based Disparities	2004	Journal of the American Medical Association
16	Yellen S.B., et al. [61]	Measuring Fatigue And Other Anemia-Related Symptoms With The Functional Assessment Of Cancer Therapy (FACT) Measurement System	1997	Journal of Pain and Symptom Management
17	Serlin R.C., et al. [75]	When Is Cancer Pain Mild, Moderate Or Severe? Grading Pain Severity By Its Interference With Function	1995	Pain
18	Portenoy R.K., et al. [55]	The Memorial Symptom Assessment Scale: An Instrument For The Evaluation Of Symptom Prevalence, Characteristics And Distress	1994	European Journal of Cancer
19	Wei J.T., et al. [76]	Development And Validation Of The Expanded Prostate Cancer Index Composite (EPIC) For Comprehensive Assessment Of Health-Related Quality Of Life In Men With Prostate Cancer	2000	Urology
20	Cassileth B.R., et al. [77]	Information And Participation Preferences Among Cancer Patients	1980	Annals of Internal Medicine
21	Degner L.F., et al. [78]	Information Needs And Decisional Preferences In Women With Breast Cancer	1997	Journal of the American Medical Association
22	Bakitas M., et al. [79]	Effects Of A Palliative Care Intervention On Clinical Outcomes In Patients With Advanced Cancer: The Project ENABLE II Randomized Controlled Trial	2009	Journal of the American Medical Association
23	Peterman A.H., et al. [59]	Measuring Spiritual Well-Being In People With Cancer: The Functional Assessment Of Chronic Illness Therapy - Spiritual Well-Being Scale (FACIT-Sp)	2002	Annals of Behavioral Medicine
24	Litwin M.S., et al. [53]	Quality-Of-Life Outcomes In Men Treated For Localized Prostate Cancer	1995	Journal of the American Medical Association
25	Fawzy F.I., et al. [80]	Malignant Melanoma: Effects Of An Early Structured Psychiatric Intervention, Coping, And Affective State On Recurrence And Survival 6 Years Later	1993	Archives of General Psychiatry
26	Schipper H., et al. [81]	Measuring The Quality Of Life Of Cancer Patients: The Functional Living Index-Cancer: Development And Validation	1984	Journal of Clinical Oncology
27	Zech D.F., et al. [82]	Validation Of World Health Organization Guidelines For Cancer Pain Relief: A 10-Year Prospective Study	1995	Pain
28	Wolfe J., et al. [83]	Symptoms And Suffering At The End Of Life In Children With Cancer	2000	New England Journal of Medicine
29	Shacham, S. [84]	A Shortened Version Of The Profile Of Mood States	1983	Journal of Personality Assessment
30	Curt G.A., et al. [58]	Impact Of Cancer-Related Fatigue On The Lives Of Patients: New Findings From The Fatigue Coalition	2000	Oncologist
31	Degner L.F., & Sloan J.A. [85]	Decision Making During Serious Illness: What Role Do Patients Really Want To Play?	1992	Journal of Clinical Epidemiology
32	Speck R.M., et al. [86]	An Update Of Controlled Physical Activity Trials In Cancer Survivors: A Systematic Review And Meta-Analysis	2010	Journal of Cancer Survivorship
33	Foley K.M. [87]	The Treatment Of Cancer Pain	1985	New England Journal of Medicine
34	Demark-Wahnefried W., et al. [88]	Riding The Crest Of The Teachable Moment: Promoting Long-Term Health After The Diagnosis Of Cancer	2005	Journal of Clinical Oncology
35	Burgess C., et al. [89]	Depression And Anxiety In Women With Early Breast Cancer: Five Year Observational Cohort Study	2005	British Medical Journal
36	Calman K.C. [90]	Quality Of Life In Cancer Patients—An Hypothesis.	1984	Journal of Medical Ethics
37	Bower J.E., et al. [91]	Fatigue In Breast Cancer Survivors: Occurrence, Correlates, And Impact On Quality Of Life	2000	Journal of Clinical Oncology
38	Reiche E.M.V., et al. [92]	Stress, Depression, The Immune System, And Cancer	2004	Lancet Oncology
39	Zimmermann C., et al. [93]	Early Palliative Care For Patients With Advanced Cancer: A Cluster-Randomised Controlled Trial	2014	The Lancet
40	Rock C.L., et al. [94]	Nutrition And Physical Activity Guidelines For Cancer Survivors	2012	CA: Cancer Journal for Clinicians
41	Spiegel D., et al. [50]	Group Support For Patients With Metastatic Cancer: A Randomized Prospective Outcome Study	1981	Archives of General Psychiatry
42	Meyer T.J., & Mark M.M. [95]	Effects Of Psychosocial Interventions With Adult Cancer Patients: A Meta-Analysis Of Randomized Experiments	1995	Health Psychology
43	Breitbart W., et al. [96]	Depression, Hopelessness, And Desire For Hastened Death In Terminally Ill Patients With Cancer	2000	Journal of the American Medical Association
44	Antoni M.H., et al. [97]	Cognitive-Behavioral Stress Management Intervention Decreases The Prevalence Of Depression And Enhances Benefit Finding Among Women Under Treatment For Early-Stage Breast Cancer	2001	Health Psychology
45	Detmar S.B., et al. [98]	Health-Related Quality-Of-Life Assessments And Patient-Physician Communication: A Randomized Controlled Trial	2002	Journal of the American Medical Association
46	De Haes M., et al. [99]	Measuring Psychological And Physical Distress In Cancer Patients: Structure And Application Of The Rotterdam Symptom Checklist	1990	British Journal of Cancer
47	McNeely M.L., et al. [100]	Effects Of Exercise On Breast Cancer Patients And Survivors: A Systematic Review And Meta-Analysis	2006	Canadian Medical Association Journal
48	Glimelius B., et al. [101]	Randomized Comparison Between Chemotherapy Plus Best Supportive Care With Best Supportive Care In Advanced Gastric Cancer	1997	Annals of Oncology
49	Goodwin P.J., et al. [102]	The Effect Of Group Psychosocial Support On Survival In Metastatic Breast Cancer	2001	New England Journal of Medicine
50	Lerman C., et al. [103]	BRCA1 Testing In Families With Hereditary Breast-Ovarian Cancer: A Prospective Study Of Patient Decision Making And Outcomes	1996	Journal of the American Medical Association
51	Vogelzang N.J., et al. [60]	Patient, Caregiver, And Oncologist Perceptions Of Cancer-Related Fatigue: Results Of A Tripart Assessment Survey	1997	Seminars in Hematology
52	Speca M., et al. [104]	A Randomized, Wait-List Controlled Clinical Trial: The Effect Of A Mindfulness Meditation-Based Stress Reduction Program On Mood And Symptoms Of Stress In Cancer Outpatients	2000	Psychosomatic Medicine
53	Molassiotis A., et al. [105]	Use Of Complementary And Alternative Medicine In Cancer Patients: A European Survey	2005	Annals of Oncology
54	Litwin M.S., et al. [54]	The UCLA Prostate Cancer Index: Development, Reliability, And Validity Of A Health-Related Quality Of Life Measure	1998	Medical Care
55	Taylor S.E., et al. [106]	Attributions, Beliefs About Control, And Adjustment To Breast Cancer	1984	Journal of Personality and Social Psychology
56	Fallowfield L., et al. [107]	Efficacy Of A Cancer Research UK Communication Skills Training Model For Oncologists: A Randomised Controlled Trial	2002	Lancet
57	Cordova M.J., et al. [62]	Posttraumatic Growth Following Breast Cancer: A Controlled Comparison Study	2001	Health Psychology
58	Gomes B., & Higginson I.J. [108]	Factors Influencing Death At Home In Terminally Ill Patients With Cancer: Systematic Review	2006	British Medical Journal
59	Slevin M.L., et al. [109]	Attitudes To Chemotherapy: Comparing Views Of Patients With Cancer With Those Of Doctors, Nurses, And General Public	1990	British Medical Journal
60	Meyerowitz B.E., & Chaiken S. [110]	The Effect Of Message Framing On Breast Self-Examination Attitudes, Intentions, And Behavior	1987	Journal of Personality and Social Psychology
61	Scheithauer W., et al. [111]	Randomised Comparison Of Combination Chemotherapy Plus Supportive Care With Supportive Care Alone In Patients With Metastatic Colorectal Cancer	1993	British Medical Journal
62	Fallowfield L.J., et al. [112]	Psychological Outcomes Of Different Treatment Policies In Women With Early Breast Cancer Outside A Clinical Trial	1990	British Medical Journal
63	Rutten L.J.F., et al. [113]	Information Needs And Sources Of Information Among Cancer Patients: A Systematic Review Of Research (1980–2003)	2005	Patient Education and Counseling
64	Leydon G.M., et al. [114]	Cancer Patients’ Information Needs And Information Seeking Behaviour: In Depth Interview Study	2000	British Medical Journal
65	McCorkle R., & Young K. [115]	Development Of A Symptom Distress Scale.	1978	Cancer Nursing
66	Riley V. [116]	Psychoneuroendocrine Influences On Immunocompetence And Neoplasia	1981	Science
67	Portenoy R.K., et al. [56]	Breakthrough Pain: Characteristics And Impact In Patients With Cancer Pain	1999	Pain
68	Brewer N.T., & Fazekas K.I. [117]	Predictors Of HPV Vaccine Acceptability: A Theory-Informed, Systematic Review	2007	Preventive Medicine
69	Kreuter M.W., et al. [118]	Achieving Cultural Appropriateness In Health Promotion Programs: Targeted And Tailored Approaches	2003	Health Education and Behavior
70	Miller G.E., et al. [119]	Chronic Psychological Stress And The Regulation Of Pro-Inflammatory Cytokines: A Glucocorticoid-Resistance Model	2002	Health Psychology
71	Hudson M.M., et al. [120]	Health Status Of Adult Long-Term Survivors Of Childhood Cancer: A Report From The Childhood Cancer Survivor Study	2003	Journal of the American Medical Association
72	Steineck G., et al. [121]	Quality Of Life After Radical Prostatectomy Or Watchful Waiting	2002	New England Journal of Medicine
73	Mulhern R.K., et al. [122]	Late Neurocognitive Sequelae In Survivors Of Brain Tumours In Childhood	2004	Lancet Oncology
74	Grunfeld E., et al. [123]	Family Caregiver Burden: Results Of A Longitudinal Study Of Breast Cancer Patients And Their Principal Caregivers	2004	Canadian Medical Association Journal
75	Ghezzi, P., et al. [124]	Impact Of Follow-Up Testing On Survival And Health-Related Quality Of Life In Breast Cancer Patients: A Multicenter Randomized Controlled Trial	1994	Journal of the American Medical Association
76	Zhang B., et al. [125]	Health Care Costs In The Last Week Of Life Associations With End-Of-Life Conversations	2009	Archives of Internal Medicine
77	Basch E., et al. [126]	Symptom Monitoring With Patient-Reported Outcomes During Routine Cancer Treatment: A Randomized Controlled Trial	2016	Journal of Clinical Oncology
78	Hann D., et al. [127]	Measurement Of Depressive Symptoms In Cancer Patients: Evaluation Of The Center For Epidemiological Studies Depression Scale (CES-D)	1999	Journal of Psychosomatic Research
79	Greer S., et al. [128]	Psychological Response To Breast Cancer: Effect On Outcome	1979	The Lancet
80	Brady M.J., et al. [57]	A Case For Including Spirituality In Quality Of Life Measurement In Oncology	1999	Psycho-Oncology
81	Jacobsen P.B., et al. [129]	Screening For Psychologic Distress In Ambulatory Cancer Patients: A Multicenter Evaluation Of The Distress Thermometer	2005	Cancer
82	Blanchard C.M., et al. [130]	Cancer Survivors’ Adherence To Lifestyle Behavior Recommendations And Associations With Health-Related Quality Of Life: Results From The American Cancer Society’s SCS-II	2008	Journal of Clinical Oncology
83	Teunissen S., et al. [131]	Symptom Prevalence In Patients With Incurable Cancer: A Systematic Review	2007	Journal of Pain and Symptom Management
84	Gøtzsche P.C., & Jørgensen K. [132]	Screening For Breast Cancer With Mammography	2013	Cochrane Database of Systematic Reviews
85	Cella, D. [52]	The Functional Assessment Of Cancer Therapy-Anemia (FACT-An) Scale: A New Tool For The Assessment Of Outcomes In Cancer Anemia And Fatigue	1997	Seminars in Hematology
86	Watson M., et al. [133]	Influence Of Psychological Response On Survival In Breast Cancer: A Population-Based Cohort Study	1999	Lancet
87	Lawlor P.G., et al. [134]	Occurrence, Causes, And Outcome Of Delirium In Patients With Advanced Cancer: A Prospective Study	2002	Archives of Internal Medicine
88	Burstein H.J., et al. [135]	Use Of Alternative Medicine By Women With Early-Stage Breast Cancer	1999	New England Journal of Medicine
89	Moorey S., et al. [136]	The Factor Structure And Factor Stability Of The Hospital Anxiety And Depression Scale In Patients With Cancer	1991	British Journal of Psychiatry
90	McClain C.S., et al. [137]	Effect Of Spiritual Well-Being On End-Of-Life Despair In Terminally-Ill Cancer Patients	2003	Lancet
91	Fawzy F.I., et al. [138]	A Structured Psychiatric Intervention For Cancer Patients: I. Changes Over Time In Methods Of Coping And Affective Disturbance	1990	Archives of General Psychiatry
92	Satin J.R., et al. [139]	Depression As A Predictor Of Disease Progression And Mortality In Cancer Patients: A Meta-Analysis	2009	Cancer
93	Novack D.H., et al. [140]	Changes In Physicians’ Attitudes Toward Telling The Cancer Patient	1979	Journal of the American Medical Association
94	Sears S.R., et al. [141]	The Yellow Brick Road And The Emerald City: Benefit Finding, Positive Reappraisal Coping, And Posttraumatic Growth In Women With Early-Stage Breast Cancer	2003	Health Psychology
95	Piper B.F., et al. [142]	The Revised Piper Fatigue Scale: Psychometric Evaluation In Women With Breast Cancer.	1998	Oncology Nursing Forum
96	Spiegel D., & Giese-Davis J. [51]	Depression And Cancer: Mechanisms And Disease Progression	2003	Biological Psychiatry
97	Helgeson V.S., & Cohen S. [143]	Social Support And Adjustment To Cancer: Reconciling Descriptive, Correlational, And Intervention Research	1996	Health Psychology
98	McNeil B.J., et al. [144]	Speech And Survival: Tradeoffs Between Quality And Quantity Of Life In Laryngeal Cancer	1981	New England Journal of Medicine
99	Stanton A.L., et al. [145]	Emotionally Expressive Coping Predicts Psychological And Physical Adjustment To Breast Cancer	2000	Journal of Consulting and Clinical Psychology
100	Doyle C., et al. [146]	Nutrition And Physical Activity During And After Cancer Treatment: An American Cancer Society Guide For Informed Choices	2006	CA: Cancer Journal for Clinicians
