# Supplementary material for: Psycho-Oncology: A Bibliometric Review of the 100 Most-Cited Articles
Source: Healthcare (Basel). 2021 Aug 6;9(8):1008. doi: 10.3390/healthcare9081008 (PMC8393329; doi:10.3390/healthcare9081008)
Supplement: Supplementary file 1 [file healthcare-09-01008-s001.zip › healthcare-1244147-supplementary.pdf]

Supplementary Material 1. Scopus String Search.

1. psycho* TITLE-ABS-KEY
2. psycho-* TITLE-ABS-KEY
3. psychosocial TITLE-ABS-KEY
4. social TITLE-ABS-KEY
5. behavio* TITLE-ABS-KEY
6. emotion*
7. quality of life TITLE-ABS-KEY
8. bereavement TITLE-ABS-KEY
9. counselling TITLE-ABS-KEY
10. coping TITLE-ABS-KEY
11. stress TITLE-ABS-KEY
12. physician-patient relationship TITLE-ABS-KEY
13. wellbeing TITLE-ABS-KEY
14. well-being TITLE-ABS-KEY
15. oncolog* TITLE-ABS-KEY
16. cancer TITLE-ABS-KEY
17. 1 OR 2 OR 3 OR 4 OR 5 OR 6 OR 7 OR 8 OR 9 0R10 OR 11 OR 12 OR 13 OR 14
18. 15 OR 16
19. 17 AND 18
20. Limit 19 to:

- English
- Journal Articles (AND (LIMIT-TO (SRCTYPE, "j"))
- 1970 on –
- AND (EXCLUDE (SUBJAREA , "BIOC") OR EXCLUDE (SUBJAREA , "PHAR") OR EXCLUDE (SUBJAREA , "CHEM")) AND (EXCLUDE (SUBJAREA , "AGRI") OR EXCLUDE (SUBJAREA , "BUSI") OR EXCLUDE (SUBJAREA , "CENG") OR EXCLUDE (SUBJAREA , "COMP") OR EXCLUDE (SUBJAREA , "ECON")) AND (EXCLUDE (SUBJAREA , "ENGI")) AND (EXCLUDE (SUBJAREA , "DENT") OR EXCLUDE (SUBJAREA , "MATH") OR EXCLUDE (SUBJAREA , "PHYS") OR EXCLUDE (SUBJAREA , "VETE") OR EXCLUDE (SUBJAREA , "ENER") OR EXCLUDE (SUBJAREA , "EART"))

Note:
TITLE-ABS-KEY = Title, abstract or keyword
BIOC = Biochemistry, Genetics and Molecular Biology
PHAR = Pharmacology, Toxicology and Pharmaceutics
CHEM = Chemistry
AGRI = Agricultural and Biological Science
BUSI = Business, Management and Accounting
CENG = Chemical Engineering
COMP = Computer Science
ECON = Economics Econometrics and Finance
ENGI = Engineering

DENT = Dentistry
MATH = Mathematics
PHYS = Physics and Astronomy
VETE = Veterinary
ENER = Energy
EART = Earth and Planetary Sciences

Supplementary Material 2. Comprehensive results from Scopus for the 100 most cited journal articles.

Rank	Authors	Title	Year	Journal
1	Aaronson N.K., et al. [69]	The European Organization For Research And Treatment Of Cancer QLQ-C30: A Quality-Of-Life Instrument For Use In International Clinical Trials In Oncology	1993	Journal of the National Cancer Institute
2	Brown K.W., & Ryan R.M. [8]	The Benefits Of Being Present: Mindfulness And Its Role In Psychological Well-Being	2003	Journal of Personality and Social Psychology
3	Temel J.S., et al. [71]	Early Palliative Care For Patients With Metastatic Non-Small-Cell Lung Cancer	2010	New England Journal of Medicine
4	Cella D.F., et al. [50]	The Functional Assessment Of Cancer Therapy Scale: Development And Validation Of The General Measure	1993	Journal of Clinical Oncology
5	Cleeland C.S., et al. [72]	Pain Assessment: Global Use Of The Brief Pain Inventory.	1994	Annals of the Academy of Medicine, Singapore
6	Taylor S.E. [73]	Adjustment To Threatening Events: A Theory Of Cognitive Adaptation	1983	American Psychologist
7	Spiegel D., et al. [49]	Effect Of Psychosocial Treatment On Survival Of Patients With Metastatic Breast Cancer	1989	The Lancet
8	Zabora J., et al. [74]	The Prevalence Of Psychological Distress By Cancer Site	2001	Psycho-Oncology
9	Sanda M.G., et al. [75]	Quality Of Life And Satisfaction With Outcome Among Prostate-Cancer Survivors	2008	New England Journal of Medicine
10	Wright A.A., et al. [76]	Associations Between End-Of-Life Discussions, Patient Mental Health, Medical Care Near Death, And Caregiver Bereavement Adjustment	2008	Journal of the American Medical Association
11	Derogatis L.R., et al. [77]	The Prevalence Of Psychiatric Disorders Among Cancer Patients	1983	Journal of the American Medical Association
12	Carver C.S., et al. [78]	How Coping Mediates The Effect Of Optimism On Distress: A Study Of Women With Early Stage Breast Cancer	1993	Journal of Personality and Social Psychology
13	Spitzer W.O., et al. [79]	Measuring The Quality Of Life Of Cancer Patients. A Concise QL-Index For Use By Physicians	1981	Journal of Chronic Diseases
14	Holmes M.D., et al. [80]	Physical Activity And Survival After Breast Cancer Diagnosis	2005	Journal of the American Medical Association

15	Murthy V.H., et al. [81]	Participation In Cancer Clinical Trials: Race-, Sex-, And Age-Based Disparities	2004	Journal of the American Medical Association
16	Yellen S.B., et al. [59]	Measuring Fatigue And Other Anemia-Related Symptoms With The Functional Assessment Of Cancer Therapy (FACT) Measurement System	1997	Journal of Pain and Symptom Management
17	Serlin R.C., et al. [82]	When Is Cancer Pain Mild, Moderate Or Severe? Grading Pain Severity By Its Interference With Function	1995	Pain
18	Portenoy R.K., et al. [54]	The Memorial Symptom Assessment Scale: An Instrument For The Evaluation Of Symptom Prevalence, Characteristics And Distress	1994	European Journal of Cancer
19	Wei J.T., et al. [83]	Development And Validation Of The Expanded Prostate Cancer Index Composite (EPIC) For Comprehensive Assessment Of Health-Related Quality Of Life In Men With Prostate Cancer	2000	Urology
20	Cassileth B.R., et al. [84]	Information And Participation Preferences Among Cancer Patients	1980	Annals of Internal Medicine
21	Degner L.F., et al. [85]	Information Needs And Decisional Preferences In Women With Breast Cancer	1997	Journal of the American Medical Association
22	Bakitas M., et al.	Effects Of A Palliative Care Intervention On Clinical Outcomes In Patients With Advanced Cancer: The Project ENABLE II Randomized Controlled Trial	2009	Journal of the American Medical Association
23	Peterman A.H., et al. [86]	Measuring Spiritual Well-Being In People With Cancer: The Functional Assessment Of Chronic Illness Therapy - Spiritual Well-Being Scale (FACIT-Sp)	2002	Annals of Behavioral Medicine
24	Litwin M.S., et al. [52]	Quality-Of-Life Outcomes In Men Treated For Localized Prostate Cancer	1995	Journal of the American Medical Association
25	Fawzy F.I., et al. [87]	Malignant Melanoma: Effects Of An Early Structured Psychiatric Intervention, Coping, And Affective State On Recurrence And Survival 6 Years Later	1993	Archives of General Psychiatry
26	Schipper H., et al. [88]	Measuring The Quality Of Life Of Cancer Patients: The Functional Living Index-Cancer: Development And Validation	1984	Journal of Clinical Oncology
27	Zech D.F.J., et al. [89]	Validation Of World Health Organization Guidelines For Cancer Pain Relief: A 10-Year Prospective Study	1995	Pain
28	Wolfe J., et al. [90]	Symptoms And Suffering At The End Of Life In Children With Cancer	2000	New England Journal of Medicine
29	Shacham S. [91]	A Shortened Version Of The Profile Of Mood States	1983	Journal of Personality Assessment
30	Curt G.A., et al.	Impact Of Cancer-Related Fatigue On The Lives Of Patients: New Findings From The Fatigue Coalition	2000	Oncologist

31	Degner L.F., & Sloan J.A. [60]	Decision Making During Serious Illness: What Role Do Patients Really Want To Play?	1992	Journal of Clinical Epidemiology
32	Speck R.M., et al. [92]	An Update Of Controlled Physical Activity Trials In Cancer Survivors: A Systematic Review And Meta-Analysis	2010	Journal of Cancer Survivorship
33	Foley K.M.	The Treatment Of Cancer Pain	1985	New England Journal of Medicine
34	Demark-Wahnefried W., et al. [93]	Riding The Crest Of The Teachable Moment: Promoting Long-Term Health After The Diagnosis Of Cancer	2005	Journal of Clinical Oncology
35	Burgess C., et al. [94]	Depression And Anxiety In Women With Early Breast Cancer: Five Year Observational Cohort Study	2005	British Medical Journal
36	Calman K.C. [95]	Quality Of Life In Cancer Patients--An Hypothesis.	1984	Journal of Medical Ethics
37	Bower J.E., et al. [96]	Fatigue In Breast Cancer Survivors: Occurrence, Correlates, And Impact On Quality Of Life	2000	Journal of Clinical Oncology
38	Reiche E.M.V., et al. [97]	Stress, Depression, The Immune System, And Cancer	2004	Lancet Oncology
39	Zimmermann C., et al. [98]	Early Palliative Care For Patients With Advanced Cancer: A Cluster-Randomised Controlled Trial	2014	The Lancet
40	Rock C.L., et al. [99]	Nutrition And Physical Activity Guidelines For Cancer Survivors	2012	CA: Cancer Journal for Clinicians
41	Spiegel D., et al. [47]	Group Support For Patients With Metastatic Cancer: A Randomized Prospective Outcome Study	1981	Archives of General Psychiatry
42	Meyer T.J., & Mark M.M. [100]	Effects Of Psychosocial Interventions With Adult Cancer Patients: A Meta-Analysis Of Randomized Experiments	1995	Health Psychology
43	Breitbart W., et al. [101]	Depression, Hopelessness, And Desire For Hastened Death In Terminally Ill Patients With Cancer	2000	Journal of the American Medical Association
44	Antoni M.H., et al. [102]	Cognitive-Behavioral Stress Management Intervention Decreases The Prevalence Of Depression And Enhances Benefit Finding Among Women Under Treatment For Early-Stage Breast Cancer	2001	Health Psychology
45	Detmar S.B., et al. [103]	Health-Related Quality-Of-Life Assessments And Patient-Physician Communication: A Randomized Controlled Trial	2002	Journal of the American Medical Association
46	De Haes M., et al.	Measuring Psychological And Physical Distress In Cancer Patients: Structure And Application Of The Rotterdam Symptom Checklist	1990	British Journal of Cancer
47	McNeely M.L., et al. [104]	Effects Of Exercise On Breast Cancer Patients And Survivors: A Systematic Review And Meta-Analysis	2006	Canadian Medical Association Journal

48	Glimelius B., et al. [105]	Randomized Comparison Between Chemotherapy Plus Best Supportive Care With Best Supportive Care In Advanced Gastric Cancer	1997	Annals of Oncology
49	Goodwin P.J., et al. [106]	The Effect Of Group Psychosocial Support On Survival In Metastatic Breast Cancer	2001	New England Journal of Medicine
50	Lerman C., et al. [107]	BRCA1 Testing In Families With Hereditary Breast-Ovarian Cancer: A Prospective Study Of Patient Decision Making And Outcomes	1996	Journal of the American Medical Association
51	Vogelzang N.J., et al. [57]	Patient, Caregiver, And Oncologist Perceptions Of Cancer-Related Fatigue: Results Of A Tripart Assessment Survey	1997	Seminars in Hematology
52	Speca M., et al. [108]	A Randomized, Wait-List Controlled Clinical Trial: The Effect Of A Mindfulness Meditation-Based Stress Reduction Program On Mood And Symptoms Of Stress In Cancer Outpatients	2000	Psychosomatic Medicine
53	Molassiotis A., et al. [109]	Use Of Complementary And Alternative Medicine In Cancer Patients: A European Survey	2005	Annals of Oncology
54	Litwin M.S., et al. [53]	The UCLA Prostate Cancer Index: Development, Reliability, And Validity Of A Health-Related Quality Of Life Measure	1998	Medical Care
55	Taylor S.E., et al. [110]	Attributions, Beliefs About Control, And Adjustment To Breast Cancer	1984	Journal of Personality and Social Psychology
56	Fallowfield L., et al. [111]	Efficacy Of A Cancer Research UK Communication Skills Training Model For Oncologists: A Randomised Controlled Trial	2002	Lancet
57	Cordova M.J., et al. [61]	Posttraumatic Growth Following Breast Cancer: A Controlled Comparison Study	2001	Health Psychology
58	Gomes B., & Higginson I.J. [112]	Factors Influencing Death At Home In Terminally Ill Patients With Cancer: Systematic Review	2006	British Medical Journal
59	Slevin M.L., et al. [113]	Attitudes To Chemotherapy: Comparing Views Of Patients With Cancer With Those Of Doctors, Nurses, And General Public	1990	British Medical Journal
60	Meyerowitz B.E., & Chaiken S. [114]	The Effect Of Message Framing On Breast Self-Examination Attitudes, Intentions, And Behavior	1987	Journal of Personality and Social Psychology
61	Scheithauer W., et al. [115]	Randomised Comparison Of Combination Chemotherapy Plus Supportive Care With Supportive Care Alone In Patients With Metastatic Colorectal Cancer	1993	British Medical Journal

62	Fallowfield L.J., et al. [116]	Psychological Outcomes Of Different Treatment Policies In Women With Early Breast Cancer Outside A Clinical Trial	1990	British Medical Journal
63	Rutten L.J.F., et al.[117]	Information Needs And Sources Of Information Among Cancer Patients: A Systematic Review Of Research (1980-2003)	2005	Patient Education and Counseling
64	Leydon G.M., et al. [118]	Cancer Patients' Information Needs And Information Seeking Behaviour: In Depth Interview Study	2000	British Medical Journal
65	McCorkle R., & Young K. [119]	Development Of A Symptom Distress Scale.	1978	Cancer Nursing
66	Riley V. [120]	Psychoneuroendocrine Influences On Immunocompetence And Neoplasia	1981	Science
67	Portenoy R.K., et al. [55]	Breakthrough Pain: Characteristics And Impact In Patients With Cancer Pain	1999	Pain
68	Brewer N.T., & Fazekas K.I. [121]	Predictors Of HPV Vaccine Acceptability: A Theory-Informed, Systematic Review	2007	Preventive Medicine
69	Kreuter M.W., et al. [122]	Achieving Cultural Appropriateness In Health Promotion Programs: Targeted And Tailored Approaches	2003	Health Education and Behavior
70	Miller G.E., et al. [123]	Chronic Psychological Stress And The Regulation Of Pro-Inflammatory Cytokines: A Glucocorticoid-Resistance Model	2002	Health Psychology
71	Hudson M.M., et al. [124]	Health Status Of Adult Long-Term Survivors Of Childhood Cancer: A Report From The Childhood Cancer Survivor Study	2003	Journal of the American Medical Association
72	Steineck G., et al. [125]	Quality Of Life After Radical Prostatectomy Or Watchful Waiting	2002	New England Journal of Medicine
73	Mulhern R.K., et al. [126]	Late Neurocognitive Sequelae In Survivors Of Brain Tumours In Childhood	2004	Lancet Oncology
74	Grunfeld E., et al. [127]	Family Caregiver Burden: Results Of A Longitudinal Study Of Breast Cancer Patients And Their Principal Caregivers	2004	Canadian Medical Association Journal
75	Ghezzi, P., et al. [128]	Impact Of Follow-Up Testing On Survival And Health-Related Quality Of Life In Breast Cancer Patients: A Multicenter Randomized Controlled Trial	1994	Journal of the American Medical Association
76	Zhang B., et al. [129]	Health Care Costs In The Last Week Of Life Associations With End-Of-Life Conversations	2009	Archives of Internal Medicine
77	Basch E., et al. [130]	Symptom Monitoring With Patient-Reported Outcomes During Routine Cancer Treatment: A Randomized Controlled Trial	2016	Journal of Clinical Oncology
78	Hann D., et al. [131]	Measurement Of Depressive Symptoms In Cancer Patients: Evaluation Of The	1999	Journal of Psychosomatic Research

		Center For Epidemiological Studies Depression Scale (CES-D)		
79	Greer S., et al. [132]	Psychological Response To Breast Cancer: Effect On Outcome	1979	The Lancet
80	Brady M.J., et al. [56]	A Case For Including Spirituality In Quality Of Life Measurement In Oncology	1999	Psycho-Oncology
81	Jacobsen P.B., et al. [133]	Screening For Psychologic Distress In Ambulatory Cancer Patients: A Multicenter Evaluation Of The Distress Thermometer	2005	Cancer
82	Blanchard C.M., et al. [134]	Cancer Survivors' Adherence To Lifestyle Behavior Recommendations And Associations With Health-Related Quality Of Life: Results From The American Cancer Society's SCS-II	2008	Journal of Clinical Oncology
83	Teunissen S.C.C.M., et al. [135]	Symptom Prevalence In Patients With Incurable Cancer: A Systematic Review	2007	Journal of Pain and Symptom Management
84	Gøtzsche P.C., & Jørgensen K.J. [136]	Screening For Breast Cancer With Mammography	2013	Cochrane Database of Systematic Reviews
85	Cella D. [51]	The Functional Assessment Of Cancer Therapy-Anemia (FACT-An) Scale: A New Tool For The Assessment Of Outcomes In Cancer Anemia And Fatigue	1997	Seminars in Hematology
86	Watson M., et al. [137]	Influence Of Psychological Response On Survival In Breast Cancer: A Population-Based Cohort Study	1999	Lancet
87	Lawlor P.G., et al. [138]	Occurrence, Causes, And Outcome Of Delirium In Patients With Advanced Cancer: A Prospective Study	2002	Archives of Internal Medicine
88	Burstein H.J., et al. [139]	Use Of Alternative Medicine By Women With Early-Stage Breast Cancer	1999	New England Journal of Medicine
89	Moorey S., et al.[140]	The Factor Structure And Factor Stability Of The Hospital Anxiety And Depression Scale In Patients With Cancer	1991	British Journal of Psychiatry
90	McClain C.S., et al. [141]	Effect Of Spiritual Well-Being On End-Of-Life Despair In Terminally-Ill Cancer Patients	2003	Lancet
91	Fawzy F.I., et al. [142]	A Structured Psychiatric Intervention For Cancer Patients: I. Changes Over Time In Methods Of Coping And Affective Disturbance	1990	Archives of General Psychiatry
92	Satin J.R., et al. [143]	Depression As A Predictor Of Disease Progression And Mortality In Cancer Patients: A Meta-Analysis	2009	Cancer
93	Novack D.H., et al.[144]	Changes In Physicians' Attitudes Toward Telling The Cancer Patient	1979	Journal of the American Medical Association
94	Sears S.R., et al. [145]	The Yellow Brick Road And The Emerald City: Benefit Finding, Positive Reappraisal Coping, And Posttraumatic	2003	Health Psychology

		Growth In Women With Early-Stage Breast Cancer		
95	Piper B.F., et al.	The Revised Piper Fatigue Scale: Psychometric Evaluation In Women With Breast Cancer.	1998	Oncology Nursing Forum
96	Spiegel D., & Giese-Davis J. [146]	Depression And Cancer: Mechanisms And Disease Progression	2003	Biological Psychiatry
97	Helgeson V.S., & Cohen S. [147]	Social Support And Adjustment To Cancer: Reconciling Descriptive, Correlational, And Intervention Research	1996	Health Psychology
98	McNeil B.J., et al. [148]	Speech And Survival: Tradeoffs Between Quality And Quantity Of Life In Laryngeal Cancer	1981	New England Journal of Medicine
99	Stanton A.L., et al. [149]	Emotionally Expressive Coping Predicts Psychological And Physical Adjustment To Breast Cancer	2000	Journal of Consulting and Clinical Psychology
100	Doyle C., et al. [150]	Nutrition And Physical Activity During And After Cancer Treatment: An American Cancer Society Guide For Informed Choices	2006	CA: Cancer Journal for Clinicians

Supplementary Material 3. Preferred Reporting Items for Systematic reviews and Meta-Analyses extension for Scoping Reviews (PRISMA-ScR) Checklist.

Section	Item	Prisma-Scr Checklist Item	Reported on Page #
		**Title**	
Title	1	Identify the report as a scoping review.	Identified as a bibliometric review on page 1
		**Abstract**	
Structured summary	2	Provide a structured summary that includes (as applicable): background, objectives, eligibility criteria, sources of evidence, charting methods, results, and conclusions that relate to the review questions and objectives.	1
		**Introduction**	
Rationale	3	Describe the rationale for the review in the context of what is already known. Explain why the review questions/objectives lend themselves to a scoping review approach.	2–3
Objectives	4	Provide an explicit statement of the questions and objectives being addressed with reference to their key elements (e.g., population or participants, concepts, and context) or other relevant key elements used to conceptualize the review questions and/or objectives.	2–3
		**Methods**	
Protocol and registration	5	Indicate whether a review protocol exists; state if and where it can be accessed (e.g., a Web address); and if available, provide registration information, including the registration number.	3

Section	Item	Prisma-Scr Checklist Item	Reported on Page #
Eligibility criteria	6	Specify characteristics of the sources of evidence used as eligibility criteria (e.g., years considered, language, and publication status), and provide a rationale.	3–5
Information sources*	7	Describe all information sources in the search (e.g., databases with dates of coverage and contact with authors to identify additional sources), as well as the date the most recent search was executed.	3
Search	8	Present the full electronic search strategy for at least 1 database, including any limits used, such that it could be repeated.	3–4
Selection of sources of evidence†	9	State the process for selecting sources of evidence (i.e., screening and eligibility) included in the scoping review.	3–5
Data charting process‡	10	Describe the methods of charting data from the included sources of evidence (e.g., calibrated forms or forms that have been tested by the team before their use, and whether data charting was done independently or in duplicate) and any processes for obtaining and confirming data from investigators.	4
Data items	11	List and define all variables for which data were sought and any assumptions and simplifications made.	5
Critical appraisal of individual sources of evidence§	12	If done, provide a rationale for conducting a critical appraisal of included sources of evidence; describe the methods used and how this information was used in any data synthesis (if appropriate).	N/A
Synthesis of results	13	Describe the methods of handling and summarizing the data that were charted.	5–6
		**Results**	
Selection of sources of evidence	14	Give numbers of sources of evidence screened, assessed for eligibility, and included in the review, with reasons for exclusions at each stage, ideally using a flow diagram.	6
Characteristics of sources of evidence	15	For each source of evidence, present characteristics for which data were charted and provide the citations.	6
Critical appraisal within sources of evidence	16	If done, present data on critical appraisal of included sources of evidence (see item 12).	N/A
Results of individual sources of evidence	17	For each included source of evidence, present the relevant data that were charted that relate to the review questions and objectives.	Supplementary material 2
Synthesis of results	18	Summarize and/or present the charting results as they relate to the review questions and objectives.	6–9
		**Discussion**	

Section	Item	Prisma-Scr Checklist Item	Reported on Page #
Summary of evidence	19	Summarize the main results (including an overview of concepts, themes, and types of evidence available), link to the review questions and objectives, and consider the relevance to key groups.	10–11
Limitations	20	Discuss the limitations of the scoping review process.	12–13
Conclusions	21	Provide a general interpretation of the results with respect to the review questions and objectives, as well as potential implications and/or next steps.	14–15
		**Funding**	
Funding	22	Describe sources of funding for the included sources of evidence, as well as sources of funding for the scoping review. Describe the role of the funders of the scoping review.	16

JBI = Joanna Briggs Institute; PRISMA-ScR = Preferred Reporting Items for Systematic reviews and Meta-Analyses extension for Scoping Reviews * Where *sources of evidence* (see second footnote) are compiled from, such as bibliographic databases, social media platforms, and Web sites. † A more inclusive/heterogeneous term used to account for the different types of evidence or data sources (e.g., quantitative and/or qualitative research, expert opinion, and policy documents) that may be eligible in a scoping review as opposed to only studies. This is not to be confused with *information sources* (see first footnote). ‡ The frameworks by Arksey and O'Malley (6) and Levac and colleagues (7) and the JBI guidance (4, 5) refer to the process of data extraction in a scoping review as data charting. § The process of systematically examining research evidence to assess its validity, results, and relevance before using it to inform a decision. This term is used for items 12 and 19 instead of "risk of bias" (which is more applicable to systematic reviews of interventions) to include and acknowledge the various sources of evidence that may be used in a scoping review (e.g., quantitative and/or qualitative research, expert opinion, and policy document).

From: Tricco AC, Lillie E, Zarin W, O'Brien KK, Colquhoun H, Levac D, et al. PRISMA Extension for Scoping Reviews (PRISMAScR): Checklist and Explanation. *Ann Intern Med*. **2018**, 169:467–473. doi: 10.7326/M18-0850.
